# Acute Coronary Syndromes: From The Laboratory Markers To The Coronary Vessels

**Published:** 2007-02-07

**Authors:** Palazzuoli Alberto, Iovine Francesca, Scali Chiara, Nuti Ranuccio

**Affiliations:** Department of Internal Medicine and Metabolic Diseases, Section of Cardiology, University of Siena, Le Scotte Hospital

**Keywords:** Coronary disease, Troponin, C-reactive protein, B-type natriuretic peptide

## Abstract

A number of “interesting” risk markers have been proposed as providing prognostic informations in acute coronary syndromes (ACS). Elevation in plasma inflammatory and necrosis biomarkers have been related to future cardiovascular events in individuals with or without prior myocardial infarction. Recently BNP and pro-BNP are entered in clinical practice to recognize patients at major risk, providing incremental information respect to the traditional markers. Together with these laboratory indexes, a few of promising laboratory markers once easily available, could become usefull in identification of patients at high risk.

Several studies evaluated many markers of platelet aggregation, endothelial dysfunction and vascular thrombosis, but it is not yet clear whether each of the proposed markers may provide incremental predictive information.

We describe, following the most studies reported in literature, the laboratory markers with potential clinical and prognostic power that could early help physicians in the identification of patients with impaired coronary disease and more narrowed coronary arteries.

## Introduction

Acute coronary syndromes without ST elevation (ACS) are heterogeneous disorders ranging from unstable angina pectoris (UA) without evidence of myocardial necrosis to myocardial infarction (MI) associated with a significant increase of troponin and isoenzime MB creatinkinase.^1^

Because of heterogeneous clinical presentation, ACS encompasses a wide range of events and different prognosis in relation to plaque type lesions and coronaric atherosclerosis diffusion. Risk assessment is based on clinical history and examination, electrocardiographic (ECG) characteristics and markers of myocardial damage, but it remains relatively inaccurate.

Recently, several biomarkers have been purposed to identify patients at high risk needing for more aggressive therapy or early angiography, to detect their risk profile and optimise the treatment.^2^ Biomarkers can provide three types of information in patients with ACS: first, they may be useful for the immediate diagnosis of disease; secondly, they can assist in risk stratification; thirdly, a biomarker can direct therapeutic decisions by monitoring course of disease and giving some prognostic informations.

**Figure 1:**
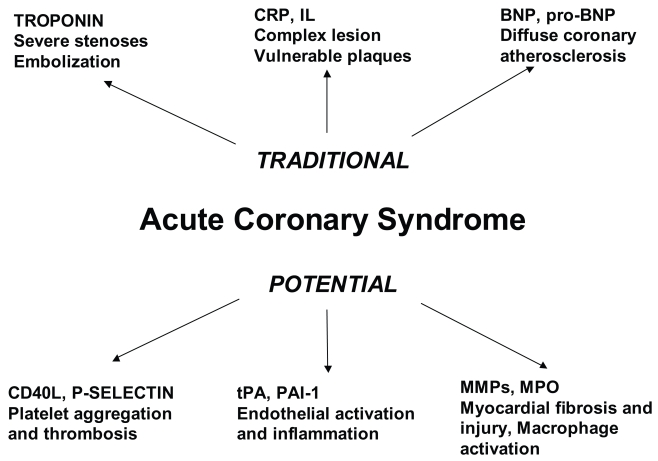
Traditional and novel laboratory risk markers and their potential clinical significance.

In this report we review, on the basis of more recent literature, the biomarkers with potential clinical and prognostic significance able to recognize patients with more advanced coronary disease; therefore, we describe the eventual possibility to identify, by laboratory research at hospital admission, patients candidates for angiographic study that would more benefit from aggressive behaviour. (figure 1)

## Pathogenesis of ACS

Advances in understanding of coronary disease have focused attention on the plaque composition rather than degree of stenosis; non stenotic lesions are more frequently implied in ACS, nonocclusive plaques may become complicated by thrombus and rapidly progress to total occlusion. Five principal causes of ACS habe been described, they include: 1) plaque rupture with acute thrombosis by formation of blood clot that gradually occludes the lumen, the pivotal event in the atherothrombotic process; 2) mechanical obstruction due to progressive increase of plaque volume; 3) inflammation, by local and sistemic production of mediators as cytokines growth factors and enzymes that activate the plaque and disrupt the fibrous cap; 4) dynamic obstruction influenced by endothelial dysfunction leading to coronary vasocostriction; 5) plaque embolization at distant level from atherothrombotic coronary occlusion.^3^,^4^ It is rare that any of these contributors exists in isolation. However, clinical presentation of ACS may vary substantially with respect to the mixture of contributions from each of these major mechanisms and patients are likely to benefit from different therapeutic strategies. The risk of subsequent death and/or recurrent ischemic events among patients with ACS also varies widely, depending on the presence or absence of irreversible myocyte injury, the hemodynamic consequences of ischemia and/or infarction, and the extent of atherosclerotic vascular disease.^5^

Traditional cardiac risk factors that predispose to atherosclerosis are only a part of the equation and must be considered within the setting of other systemic and local contributors that may adversely affect coronary plaque progression and thrombotic complication. The efforts must be addressed in early detection of culprit plaque, by more sensitive serum markers assessment to find and treat the vulnerable patients at elevated risk.

## Traditional Risk Markers

Recently AHA/ACC have purposed a TIMI risk score for identification of patients with major risk, however this classification is not completely able to identify all high risk subjects. Hence, the introduction of other markers easily available could bring to worth a better risk profile evaluation.^6^

Classical cardiac markers usually considered as strong predictors among patients with coronary disease, are Troponin T (TnT) and C-reactive Protein (CRP), both related to increased risk of recurrent ischemic events and cardiovascular death.

Elevated TnT levels appear related to more ‘active’ lesions, which in turn are associated with increased long-term coronary instability. Angiographic studies have shown an association between increased troponin levels and complex lesions, more severe coronary artery disease, decreased coronary blood flow and greater activation of the coagulation system compared to patients with normal troponin levels.^7^

Recently, a substudy from GUSTO-IV clearly demonstrated that TnT is associated with early and late risk of mortality and myocardial infarction, probably due to greater microembolization risk or to major extension of coronary atherosclerosis.^8^ Hence, the persistence of increased risk over time may reflect more severe disease with greater likelihood of continued coronary plaques instability.

The correlation between elevated CRP levels and prognosis in coronary disease may be mediated by the vascular effects of proinflammatory cytokines, including tumor necrosis factor TNF-α, interleukin IL-1 and IL-6, which induce the hepatic production of CRP.^9^ Mononuclear cells of patients with recurrent unstable angina show an enhanced production of IL-6 in response to low-dose of lipopolysaccharide (LPS), correlated with baseline CRP levels, six months after the last acute event: this persisting acute-phase responsiveness could explain the association between CRP and acute coronary events.^10^

Some works have indicated that the protein doesn’t reflect only inflammation stage that concur to atherosclerotic process, but it could have a direct role in the plaque rupture: unstable atherosclerotic plaques have an increased number of macrophages that seem to be most abundant in the vulnerable shoulder region of the fibrous cap, overlying the core of atheroma within the vessel wall.^11^ The pronounced elevation of CRP is transient and likely related to an acute-phase reaction. Some patients with ACS might have a hyperresponsiveness of the inflammatory system, which might exaggerate the acute-phase reaction and increase the immune system reaction. Such mechanism is supported by the observations of co-localization of CRP and activated complement in infarct-related myocardium. It has been shown that CRP also stimulates production of tissue factor by mononuclear cells, the main initiator of blood coagulation.^12^

CRP increase appears related to incidence of revascularization in patients with ACS and increased risk of recurrent ischemia. However, studies are not in accordance: Toss et al. recruited no correlation; on the other hand, Liuzzo et al. showed worse prognosis in patients with UA.^13^,^14^ Moreover, ECAT Angina Pectoris Study showed that elevated CRP levels highly correlate with low left ventricular ejection fraction.^15^

The highest values in CRP have been found in patients with the largest increase in troponin concentrations. High CRP levels are independent predictors of death in these patients, but are not associated with a greater risk of MI. These apparently contradictory findings may be explained by hypothesizing a strong influence of troponin values on CRP increase in ACS.^16^

## B-Type Natriuretic Peptide as Additional Risk Marker

Many studies have been shown that elevation of BNP as well as NT-proBNP levels obtained after the acute phase (median, 40 to 72 hours after symptoms onset) in patients with a broad range of ACS independently predicts mortality.^17^–^19^ However, in most of the reports, authors do not distinguish subjects with low ejection fraction and LV enlargement; in all studies, BNP levels appear related to increase of heart failure, predicting of cardiac remodeling and pump dysfunction. Recently, BNP has been related with coronary vessels involvement showing a significant correlation with left descending artery disease and TIMI flow.^20^ In another report, BNP appeared associated with multivessels disease in ACS patients without systolic dysfunction.^21^

Data from both experimental and clinical studies suggest that the plasma levels of BNP may reflect the size or severity of ischemic insult, even in absence of myocardial necrosis. Specifically, in human models of ischemia, BNP levels have been shown to increase transiently both after exercise in patients with stable coronary disease and after uncomplicated coronary angioplasty, despite stable intracardiac filling pressures.^22^,^23^ Others have shown the rise in the level of BNP is correlated with the size of an ischemic territory during nuclear stress imaging.^24^

All these data suggest that BNP could represent not only a marker of LV enlargement but also a marker of coronary disease extension: BNP could increase during ischemic damage in patients with CAD, suggesting that elevated levels are associated with larger extent as well as greater severity of ischemia, independently on LV systolic dysfunction. (figure 1)

## Biomarkers of Platelet Activation

Biomarkers of platelet activation may provide clues regarding the extent of platelet activation in patients with ACS and predict the severity of the inflammatory response. Rupture or erosion of an atheromatous plaque exposes subendothelial proteins such as collagen and von Willebrand factor, leading to adhesion via the platelets surface receptors. This adhesion causes platelet activation which triggers a conformational change in the platelet glycoprotein IIb/IIIa receptor and generates the enzyme thrombin from circulating prothrombin which converts fibrinogen to fibrin. Fibrin and von Willebrand factor bind to the GP IIb/IIIa receptor and trap multiple activated platelets leading to their aggregation.

Soluble CD40 ligand is a transmembrane glycoprotein of the tumor necrosis factor family. CD40L has been shown to promote atherosclerosis and plaque instability,^25^ to retain inflammatory effects through promotion of platelet-monocyte aggregates and production of reactive oxygen species.^26^ In addition to inflammatory properties, platelet CD40L stabilizes platelet-platelet aggregates, target of numerous antiplatelet agents used in the treatment of ACS.^27^

In patients with ACS, sCD40L identifies a subgroup at increased 6-month risk of death or non-fatal MI.^28^ The (OPUS)-TIMI 16 trial has shown that sCD40L provides prognostic ability independent of TnI or C-reactive protein.^29^ These results confirmed that ACS patients with elevated sCD40L are at higher risk of adverse coronary events relative to patients with ACS and no elevation of sCD40L.

P-selectin is a transmembrane cell adhesion molecule stored in endothelial cells and in platelet granules. Endothelial cell P-selectin mediates tethering and rolling of leukocytes along the activated endothelium, whereas platelet P-selectin mediates formation of platelet aggregates in pulsatile high-shear stress conditions.^30^ The majority of soluble P-selectin appears to be derived from platelets, because soluble P-selectin levels are correlated with established platelet markers but not with endothelial markers.

Attempts to use plasma P-selectin for risk stratification in ACS have yielded conflicting results. Some studies have suggested that P-selectin may be useful in risk stratification for patients presenting with chest pain;^31^ Yazici et al. have recently demonstrated a straight correlation between P-selectin and troponin suggesting a specific role of increased coagulation and myocardial injury. Given its possible pathophysiological role in platelet aggregation and thrombosis, P-selectin remains a possible therapeutic target.^32^

Tissue plasminogen activator (t-PA) is a glycoprotein produced mainly by vascular endothelial cells.^33^,^34^ It activates clot dissolution in the presence of fibrin by conversion of plasminogen to plasmin, thereby cleaving cross-linked fibrin to D-dimer and other degradation products and it may also be involved in coronary plaque rupture.^35^

A combined analysis of studies evaluating t-PA in coronary diseases, adjusted for some risk factors, suggests that CHD risk is about 50% greater in those with increased levels: a comparison of those in the top third with those in the bottom third of baseline t-PA antigen values yielded an odds ratio for CHD of 2.20 (1.70 – 2.85; 21 = 36) after adjustment for age and town only, and of 1.48 (1.09 – 2.01; 21 = 6) after further adjustment for classical CHD risk factors.^36^,^37^

Epidemiological studies of t-PA antigen consistently show strong correlations with PAI-1 activity or antigen.^38^ This association may reflect simultaneous release from endothelial cells, delayed clearance of t-PA-PAI-1 complexes, acute-phase reactions, or mutual correlations during early and late phases of atherosclerosis.^39^

**Figure 2:**
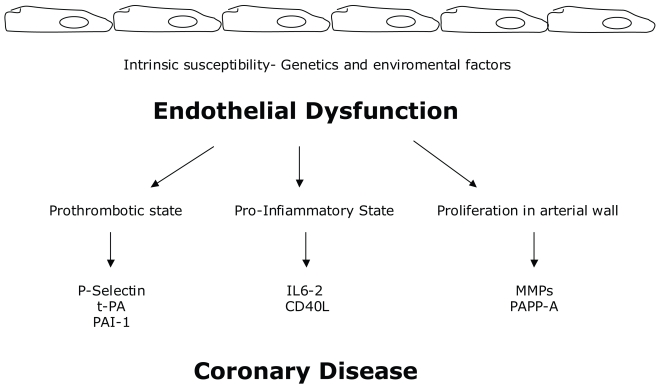
Biomarkers of endothelial dysfunction and their specific actions in atherosclerotic process.

What is the potential biological significance of the positive association between circulating t-PA antigen and risk is still unknown: t-PA is released from vascular endothelium, hence increased circulating levels may be a marker of endothelial disturbance. In one study, circulating t-PA antigen correlated strongly with circulating levels of von Willebrand factor (vWF), another endothelial release product, as well as with risk markers associated with endothelial dysfunction. Tissue plasminogen activator and PAI-1 may partly reflect their mutual association with the inflammatory response. While increased circulating free t-PA might increase fibrin lysis, no significant association of t-PA antigen was observed with fibrin D-dimer levels.^40^ (figure 2)

## Future Potential Markers

Metalloproteinases (MMPs) are zinc-dependent endoproteases with collagenase and/or gelatinase activity. Degradation of collagen fibrils compromises plaque stability and the integrity of the endothelial basament membrane, predisposing advanced atheromas to rupture.^41^

There are many types of MMPs, the most known are MMP-2, a gelatinase capably to degrade type IV collagen, represented in the subendothelial basement membrane. MMP-8 is a collagenase produced by neutrophils, but also by endothelial cells, smooth muscle cells and macrophages in plaques. MMP-9 is a gelatinase widely implicated in ventricular remodeling and the development of heart failure.^42^ MMPs are higly expressed in atheroslerotic plaques and subjects with acute coronary syndrome (ACS) have increased plasma levels of MMP-1, -2 and -9.

Some groups have found that MMP-1, -2 and -9 are not elevated at the presentation, but that they increase during the subsequent 7 to 14 days.^43^ Other groups have reported no significant elevation in MMP-2, but a rapid rise and fall of MMP-9 within the first week after ACS. Some authors measured MMP-8 concentrations in 181 patients positive for coronary artery disease (CAD) after angiography and they found that these subjects had higher MMP-8 concentrations, compared with patients without CAD, increasing with the number of stenotic vessels.^44^

Another study reports an association between MMP levels during ACS and cardiovascular outcomes. In a study of 24 patients with ACS, elevation in MMP-1 at 7 and 14 days after ACS were negatively correlated with left ventricular ejection fraction.^45^ Thus, the slow elevation of MMP levels after ACS and the lack of clinical outcome data do not currently make MMPs useful biomarkers in ACS.

It has recently recognized that polymorphonuclear neutrophils (PMNs) are critical mediators in ACS, because of their recruitment and activation before platelet aggregation.^46^ One of the principal mediators secreted on PMN activation is myeloperoxidase (MPO), a hemoproteine that is a microbicidal enzyme, but it has also potent proatherogenic properties: infact, MPO can oxidize LDL cholesterol, amplificating uptake by macrophages and perpetuating foam cell formation.^47^ Moreover, MPO promotes activation of metalloproteinases and destabilization and rupture of atherosclerotic plaques surface.^48^ Furthermore, MPO catalytically consumes endothelium-derived nitric oxide, reducing its bioavailability and impairing its vasodilatory and anti-inflammatory functions.^49^

Elevated MPO plasma levels in patients with unstable angina and acute myocardial infarction have been shown. Biasucci et al. demonstrated that neutrophils are activated in acute coronary syndromes, may be not only secondary to ischemia-reperfusion injury.^50^

Some authors found that MPO serum levels were equally distributed among patients with low and high Troponin T (TnT) serum levels, which indicates that elevated MPO levels are not temporary related to myocardial injury.^47^ Moreover, MPO identifies patients at risk for cardiovascular events who have low baseline TnT levels, even before complete microvascular obstruction. In patients with low CRP levels, MPO identifies those with increased risk for cardiovascular events. These data suggest that recruitment and degranulation of PMNs is a primary event and is followed by release of other systemic mediators and acute-phase proteins such as CRP.^46^,^49^

Pregnancy-associated plasma protein A (PAPP-A) is a peptide specifically elevated in pregnancy, specially in the first trimester and it is used for the screening of chromosomal abnormalities. PAPP-A is a zinc-binding metalloprotease that indirectly activates insuline-like growth factor (IGF), a potent mitogen and chemotactic agent for vascular smooth muscle cells. Because inhibition of IGF signaling has been shown to delay atherosclerosis, it is possible that PAPP-A indirectly promotes atherosclerosis or restenosis by increasing the activity of IGF. Infact, PAPP-A levels after angioplasty have been implicated as a possible mechanism for restenosis.^51^

In the same study has been found that PAPP-A was not associated with TnT or creatine-kinase-MB elevation, suggesting that PAPP-A is not useful for detectable myocardial infarction, though it may have diagnostic value for identifying patients with ACS. Other studies would confirm the hypothesis that PAPP-A is an independent predictor of cardiovascular events, regardless of troponin status.^52^,^53^

These studies suggest that PAPP-A may define subsequent cardiovascular risk in patients with unstable angina and no troponin elevation. Moreover, increased serum levels of PAPP-A may reflect instability of atherosclerotic plaques.^54^

In the last few years, several studies demonstrated that raised levels of interleukin-6 are common in unstable angina, correlate with C-reactive protein, and are associated with prognosis, thus confirming the importance of the cytokine pathway for the production by the liver of acute-phase proteins and strengthening the importance of inflammation in this syndrome. Therefore, interleukin (IL)-18, originally identified as an interferon (IFN)-γ-inducing factor in Kupffer cells and macrophages, has been proved to play a central role in the inflammatory cascade. Furthermore, IL-18 acts in synergy with IL-12 to promote the development of T helper 1 responses.^55^ Recently, increased IL-18 expression has been reported in human atherosclerotic plaque, mediating INF-γ release locally. Moreover, animal models support the proatherogenic role of IL-18 as well as the beneficial effect of inhibiting IL-18 on plaque progression and composition. Thus, serum IL-18 level has been recently identified as a strong independent predictor of death from cardiovascular causes in patients with coronary artery disease regardless of the clinical status at admission. This result strongly supports recent experimental evidence of IL-18-mediated inflammation leading to acceleration and vulnerability of atherosclerotic plaques.^56^

The ST2 gene is a member of the interleukin-1 receptor host defense-inflammation family. The protein product of ST2 encodes a transmembrane receptor form (ST2L) and a truncated soluble receptor form (ST2) that can be detected in human serum. It is known that ST2 directs Th2 lymphocyte function; this finding suggests that ST2 is induced in conditions of myocardial overload such as myocardial infarction, when the remaining viable myocardium must bear more stress. Indeed, soluble ST2 levels are increased in the serum of patients one day after myocardial infarction. Furthermore, ST2 serum levels predict outcome in patients with heart failure, and a change in ST2 over time is also associated with prognosis.^57^ These data suggest that serum levels of the interleukin-1 receptor family member ST2 predict mortality and heart failure in patients with acute myocardial infarction.^58^

## Conclusions

Several emerging laboratory markers are now disposal for diagnosis and prognosis of ACS. Some common parameters are actually recommended for risk stratification (Troponine, CRP and BNP or pro-BNP), some others could become important to identify patients at higher risk: the more promising seem to be parameters of platelet activation (P-selectin and t-PA) and inflammatory markers mediators (MPO) (figure 1).

Every cited markers could represent a specific index of atherosclerotic process and plaque activation, physicians effort could address in the discover and identification of laboratory parameters with both easy sample and clinical significance application. The combination approach could permit in the future to encompass from the laboratory to the clinical aspects and to recognize patients with more dangerous coronary lesions and coronary disease severity.
